# Is Diffusion Tensor Imaging a Good Biomarker for Early Parkinson's Disease?

**DOI:** 10.3389/fneur.2018.00626

**Published:** 2018-08-21

**Authors:** Rachel P. Guimarães, Brunno M. Campos, Thiago J. de Rezende, Luiza Piovesana, Paula C. Azevedo, Augusto C. Amato-Filho, Fernando Cendes, Anelyssa D'Abreu

**Affiliations:** ^1^Department of Neurology, University of Campinas, Campinas, Brazil; ^2^Laboratory of Neuroimaging, University of Campinas, Campinas, Brazil; ^3^Laboratory of Medical Physics, University of Campinas, Campinas, Brazil; ^4^Department of Radiology, University of Campinas, Campinas, Brazil

**Keywords:** Parkinson's disease, movement disorders, neuroimaging, diffusion tensor imaging, magnetic resonance imaging

## Abstract

**Objectives:** To assess white matter abnormalities in Parkinson's disease (PD).

**Methods:** A hundred and thirty-two patients with PD (mean age 60.93 years; average disease duration 7.8 years) and 137 healthy controls (HC; mean age 57.8 years) underwent the same MRI protocol. Patients were assessed by clinical scales and a complete neurological evaluation. We performed a TBSS analysis to compare patients and controls, and we divided patients into early PD, moderate PD, and severe PD and performed an ROI analysis using tractography.

**Results:** With TBSS we found lower FA in patients in corpus callosum, internal and external capsule, corona radiata, thalamic radiation, sagittal stratum, cingulum and superior longitudinal fasciculus. Increased AD was found in the corpus callosum, fornix, corticospinal tract, superior cerebellar peduncle, cerebral peduncle, internal and external capsules, corona radiata, thalamic radiation and sagittal stratum and increased RD were seen in the corpus callosum, internal and external capsules, corona radiata, sagittal stratum, fornix, and cingulum. Regarding the ROIs, a GLM analysis showed abnormalities in all tracts, mainly in the severe group, when compared to HC, mild PD and moderate PD.

**Conclusions:** Since major abnormalities were found in the severe PD group, we believe DTI analysis might not be the best tool to assess early alterations in PD, and probably, functional and other structural analysis might suit this purpose better. However it can be used to differentiate disease stages, and as a surrogate marker to assess disease progression, being an important measure that could be used in clinical trials.

**HIGHLIGHTS**

- DTI is not the best tool to identify early PD
- DTI can differentiate disease stages
- DTI analysis may be a useful marker for disease progression

## Introduction

Parkinson's disease (PD) is classically defined by dopamine dysfunction, and the diagnosis is clinical, made in the presence of at least one motor symptom such as bradykinesia, rest tremor, rigidity and postural instability (1). Pathological studies describe that synuclein is first found at lower brainstem and or olfactory bulb (2, 3), corroborating the idea that brain affection goes beyond the motor system, and that nondopaminergic neurons are also involved (4).

PD is mainly known for its motor symptoms; however, non-motor symptoms have a significant impact in patient's quality of life. Cognitive impairments are common in PD patients and can be present at initial diagnosis, though they are more frequent at late disease stages (5, 6).

The development of a neuroimaging biomarker for early PD diagnosis is critical and might have a high impact on patients' quality of life. Diffusion tensor imaging (DTI) is a valuable tool to assess white matter (WM) abnormalities, and it can be used in the longitudinal assessment of patients as well as markers of disease progression and treatment response (7, 8).

The measures obtained from DTI are fractional anisotropy (FA), mean diffusivity (MD), radial diffusivity (RD) and axial diffusivity (AD). FA quantifies the preferred diffusion direction of water molecules through WM tracts, and MD represents diffusion magnitude (9, 10). More recent studies suggest that eigenvalues amplitude might have a better relation to WM abnormalities, as AD relates to axonal degeneration and RD seems to be modulated by the presence of myelin (10). Thus, diffusion-weighted analysis can demonstrate neuronal/axonal loss through altered diffusion values.

Previous studies in DTI still lack essential information about alteration beyond the substantial nigra (SN), which is, according to a recent meta-analysis (11) the main consensual information related to the neurodegenerative process in PD; however, its association with disease severity is still unclear (12). One study associated WM lesions with cognitive impairment (13), while alterations in olfactory tracts and anterior olfactory structures have also been previously reported, in accordance to olfactory complaints in PD patients and previous findings of reduced cortical thickness in the olfactory cortex in PD (14).

A meta-analysis showed that DTI is sensitive in detecting differences between PD and HC, particularly in the SN, the corpus callosum, cingulate and temporal cortices, and the corticospinal tract (11).

Our purpose was to perform exploratory DTI analysis with TBSS and then focus on tracts known to be relevant to PD, but that still lack information about their real role in PD pathophysiology.

## Methods

We conducted a cross-sectional study at the Neuroimaging Laboratory—University of Campinas (Unicamp) Hospital. The Unicamp Ethics Committee approved the study, and all individuals signed an informed written consent prior to any research-related procedure.

### Subjects

All individuals were recruited at the Movement Disorders Outpatient Clinic at the University Hospital-UNICAMP. 132 patients, age over 30 years old (85 men, mean age 61 years, SD 10.04; average disease duration 7.8, SD 6.67) previously diagnosed with PD according to the London Brain Bank criteria, underwent MRI. Ninety-seven (97) were assessed by clinical scales. All patients were assessed and scanned on medication. An experienced neurologist specialized in Movement Disorders assessed all patients. Evaluation consisted of a standardized questionnaire regarding sex, age, age at disease onset, family history, professional history, environmental exposure to risk factors and medication used, as well as the application of the Unified Parkinson's Disease Rating Scale (UPDRS), Hoehn and Yahr scale (H&Y), Scales for Outcomes in Parkinson's disease—Cognition (SCOPA-COG), and (SCOPA-PC), SCHWAB&ENGLAND activities of daily living and Non-motor symptoms scale (NMSS). We also selected 137 healthy controls (HC) (83 men, mean age 58, SD 9.39) with a normal neurologic examination and no history of neurologic or psychiatric disease, or a family history of PD. HC were recruited at the hospital and were non-consanguineous relatives of the patients or employees from the hospital. There was no difference between PD and HC regarding age and sex (*p* = 0.08).

**Figure 1:**
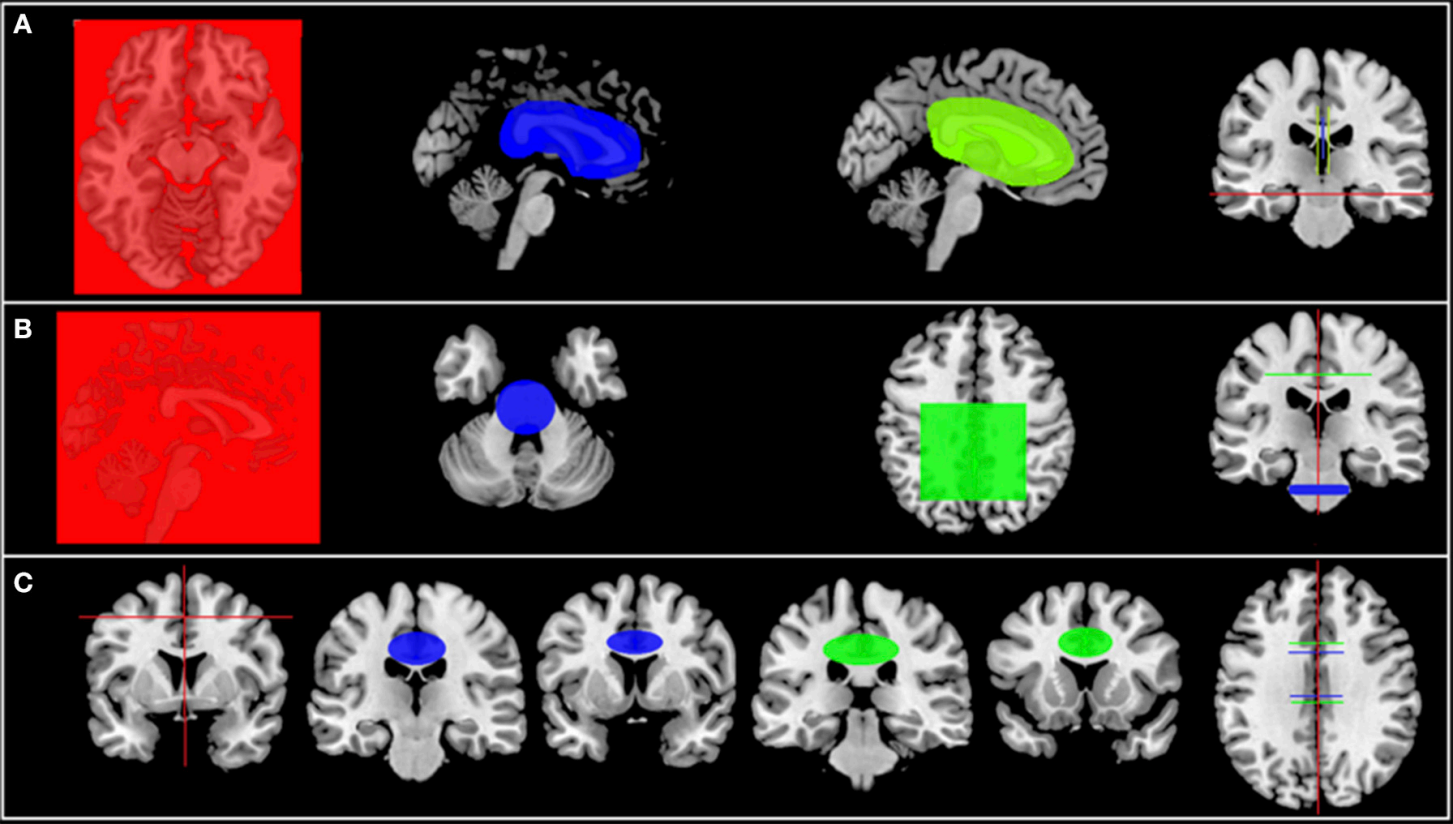
**(A-C)** Illustration of the adopted strategy for each tract studied.

We compared all PD patients with HC using TBSS. Then we performed a region of interest (ROI) analysis, dividing patients according to disease severity into mild PD (MPD) (H&Y 1–1.5) (*n* = 24), moderate PD (MoPD) (H&Y 2–3) (*n* = 60) and severe PD (SPD) (H&Y 4–5) (*n* = 13) and made comparisons within groups in the corticospinal tract (CST), cingulum and corpus callosum (CC) (Figure 1).

### MRI acquisition

All subjects underwent the same protocol for MRI acquisition on a 3T Philips Achieva MRI scanner at Unicamp that, in addition to the structural imaging, included diffusion-weighted images with 32 directions: 2 mm isotropic voxel, time of repetition (TR), 8500; time of echo (TE), 61; b-factor, 1,000; matrix 128 × 127; and field of view (FOV), 256 × 256.

### Tract-based spatial statistics

For processing the DTI, all imaging data were transferred to a cluster of Linux workstations. The structural images were visually inspected for any structural abnormalities by a neuroimaging expert. Preprocessing and analyses of diffusion data were done with an in-house protocol using FMRIB Software Library (FSL, version 5.0). We used the raw image for performing offline registration and motion correction.

Diffusion-weighted images were analyzed using FFSL tract-based spatial statistics (TBSS) (15) via the following routine. Firstly, the FDT toolbox was used to correct all data for spurious eddy current distortions and motion artifacts by applying affine alignment of each diffusion-weighted image to the first volume of the diffusion data without gradient (i.e., the b = 0 image). The Brain Extraction Tool (BET) was then used to generate a binary brain mask from the b = 0 image. Next DTIfit was used to independently fit the diffusion tensor to each voxel which yielded voxel-wise maps of FA values. The statistical analysis was done using a two-sample *t*-test to look for differences between patients and controls regarding FA, MD, AD, and RD parameters. We used a threshold-free cluster enhancement (TFCE) to correct for multiple comparisons (*p* < 0.05).

For localizing the significant results, we used the Johns Hopkins WM DTI atlas offered by FSL (http://www.fmrib.ox.ac.uk/fsl/data/atlas-descriptions.html#wm).

### Region of interest analysis

The DTI images were corrected for subject motion using Philips Registration Algorithms during the image reconstruction, generating an intrasubject registered image. The images were corrected for signal drifts using the appropriate Explore DTI plugin. The diffusion tensor was estimated using Explore DTI Weighted Linear algorithms, all processed images and fiber tracts were visually inspected. Also, as we mentioned in the TBSS methods section, all structural images were visually inspected for any structural abnormalities by a neuroimaging expert. The same step was performed for the ROI analysis.

The fiber tractography was performed through a semiautomatic deterministic methodology briefly described later (16). ROIs to seed the tract were manually drawn on the MNI-152 template and were based on DTI images of 10 Brazilian subjects (5 females, age, 22–47 years, average 33 years) (Figure 1). Sequentially, the method uses the three-dimensional (3D) deformation field matrix of each subject to apply an inverse normalization operation (Statistical Parametric Mapping 8 [SPM 8]-deformation fields algorithm), using the variants between native and standardized space to bring the normalized ROIs to that subject-specific space. Finally, the adjusted (native space) ROIs were used for the fiber tracking.

The fiber-tracking parameters set were: minimal FA to start tract 0.25; minimal FA to keep tracking 0.25; maximal tract angle of 60°; and minimal fiber length 10 mm. We visually checked the resultant tracts and separately calculated the average FA, AD, and RD for each hemisphere. The diffusion values were estimated by averaging all voxels in a given tract.

For statistical analysis, we used Stata software, version 13.1 (http://www.stata.com).

To find differences in FA, AD, and RD values between groups, we performed a GLM for each tract, including FA, AD, and RD as dependent variables, and subgroups (MPD, MoPD, and SPD) as the independent variables and regressed out sex and age.

We also employed a GLM to investigate the correlation of FA, AD, and RD results with clinical parameters (UPDRS, UPDRS-III, NMSS, and SCOPA-COG). We set a *p*-value of 0.05 for significant results, with Sidak correction for multiple comparisons.

## Results

We compared 132 patients and 137 controls. We had complete clinical data from 97 patients. Patients information are described in Table 1.

**Table 1:** Patients demographic data, mean ± standard deviation.

	**ALL PD**	**MPD**	**MoPD**	**SPD**
Age	60.93 ± 9.8	61.66 ± 9.6	60.30 ± 10.69	61.96 ± 8.4
Time of disease	7.8 ± 6.43	4.15 ± 4.08	8.74 ± 6.62	12.14 ± 5.31
UPDRS	34.4 ± 18.47	20.16 ± 8.66	35.38 ± 13.38	59.71 ± 17.15
UPDRS-PartIII	16 ± 8.23	10.92 ± 4.78	16.33 ± 5.95	26.71 ± 8.88
H&Y	2.8 ± 1.26	1.5 ± 0.25	3 ± 0.3	4.3 ± 0.5
SCOPA PC	3.05 ± 7.8	1.86 ± 1.72	2.81 ± 2.37	6 ± 18.63
SCOPA	17.7 ± 6.8	20.7 ± 3.77	17.43 ± 7.18	14.14 ± 7.15
NMSS	67.4 ± 48.19	36.44 ± 34.97	70 ± 46.95	92.92 ± 52.43
Education	6.7 ± 4.7	8.44 ± 4.3	6.4 ± 5.01	4.15 ± 3.3
Time of medication	6.9 ± 5.6	2.5 ± 2.42	6.13 ± 6.2	9.4 ± 5.75

*Parkinson's Disease (PD), Mild Parkinson's Disease (MPD), Moderate Parkinson's Disease (MoPD), Severe Parkinson's Disease (SPD), Unified Parkinson's Disease Rating Scale (UPDRS), Hoehn Yahr (H&Y), Scale for Outcomes in Parkinson's Disease – Psychiatric Complications (SCOPA-PC), Scale for Outcomes in Parkinson's Disease – Cognition (SCOPA-COG) Non Motor Symptoms Scale (NMSS)*.

## Tbss

**Figure 2:**
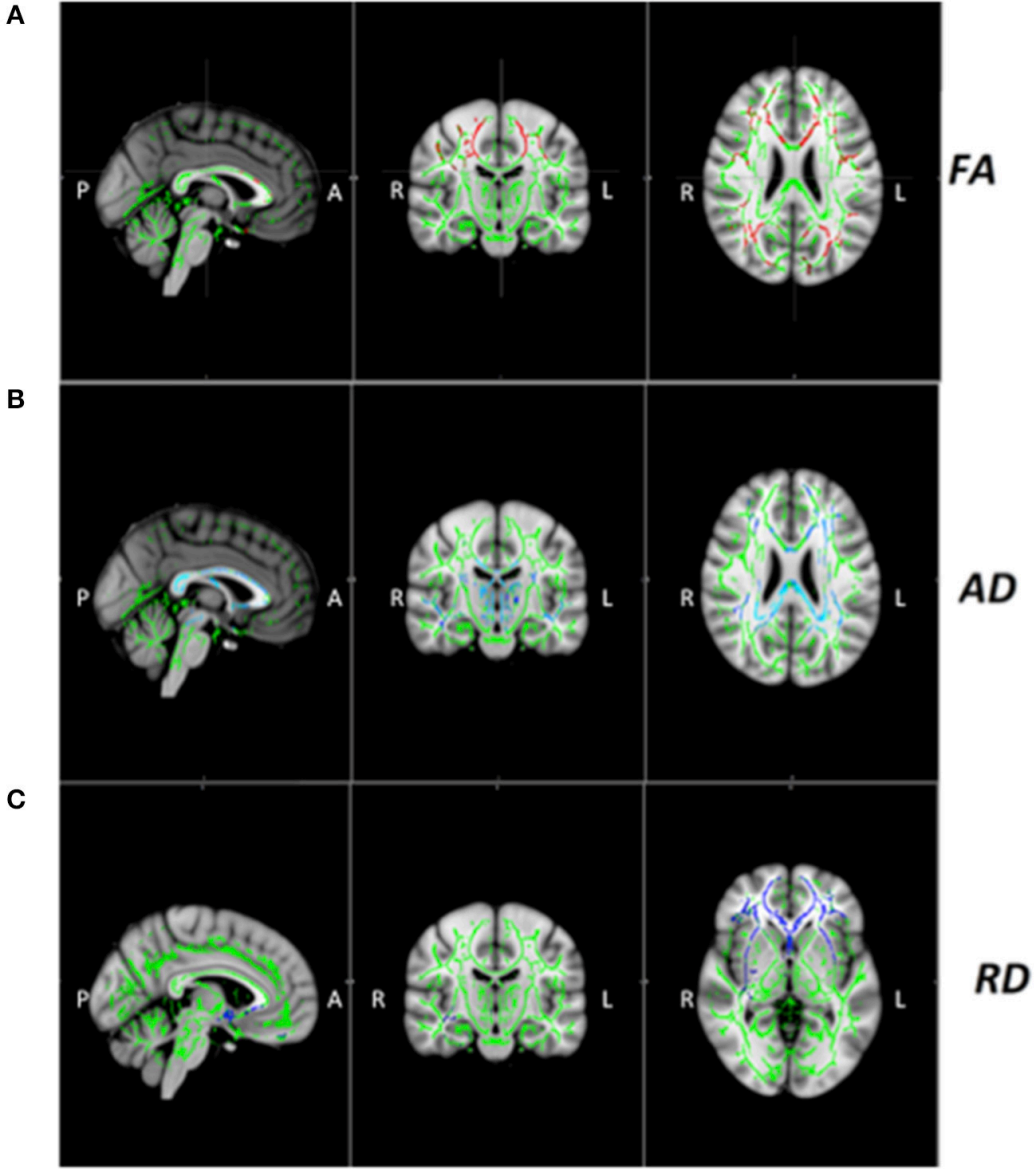
**(A-C)** White matter diffusion tensor MRI findings in patients with Parkinson's Disease (PD). Decreased fractional anisotropy (FA, red) (*x* = 91, *y* = 109, *z* = 97 MNI coordinates) and increased axial (AD, blue) (x = 93, *y* = 109, *z* = 97) and radial diffusivities (RD, blue) (*x* = 87, *y* = 109, *z* = 66) in patients PD relative to healthy controls (HC).Result are overlaid on the Montreal Neurological Institute standard brain, and displayed at *p* < 0.05 corrected for multiple comparisons at the cluster level using the threshold-free cluster enhancement option. The white matter skeleton is green. L, left; R, right.

Voxelwise statistical comparison between PD and HC showed significantly lower FA in patients in genu, body, and splenium of corpus callosum, internal and external capsule, corona radiata, posterior thalamic radiation, sagittal stratum, cingulum and superior longitudinal fasciculus (Figure 2A). Increased AD was found in genu, body, and splenium of corpus callosum, fornix, corticospinal tract, superior cerebellar peduncle, cerebral peduncle, internal and external capsules, corona radiata, thalamic radiation and sagittal stratum (Figure 2B). Increased RD was seen in the genu of corpus callosum, internal and external capsules, corona radiata, sagittal stratum, fornix, and cingulum (Figure 2C).

## ROI analysis

### 1- corticospinal tract

There was no FA difference between groups. SPD had higher AD in relation to MPD, and higher RD values in relation to HC (*p* < 0.01).

There was a negative association between SCOPA-COG scores and AD values and a positive association between AD and UPDRS and UPDRS-III scores (*p* < 0.01).

### 2- cingulum

There was no FA difference between groups. AD and RD were higher in SPD when compared to HC, MPD and MoPD (*p* < 0.01).

There was a positive association between SCOPA-COG scores and FA values, and a negative association with RD. UPDRS, UPDRS-III and NMSS, were positively associated with AD and RD values (*P* < 0.01).

### 3- corpus callosum

SPD had lower FA in relation to HC and MPD (*p* < 0.01). They also had higher RD and AD values in comparison to HC, MPD and MoPD (*p* < 0.01).

**Figure 3:**
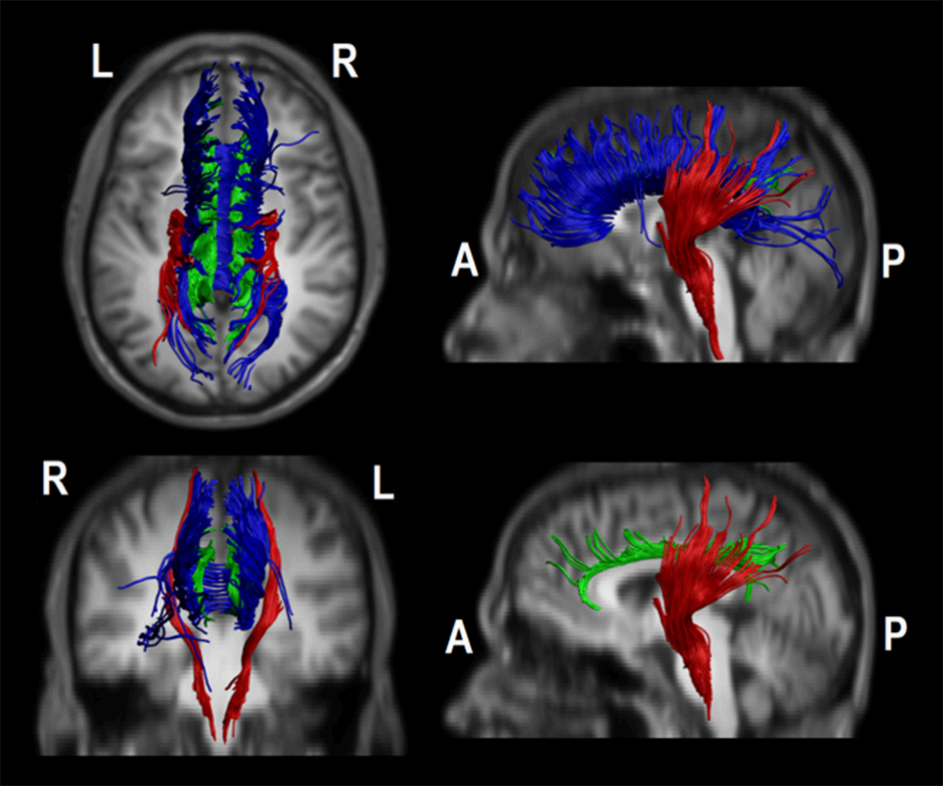
The figure represent all studied tracts in a representative controls object. The tracts were drawn on native space overlapping a T1 W1 spatially transformed to match to the DT1 native space (voxel size: 1 × 1 × 2 mm^3^; Matrix: 256 × 256 × 70). In blue the body of the corpus callosum, in green the body of the cingulum and in red an example of the corticospinal tract. The bottom-right image hides the body of the corpus callosum to better illustrate in a sagittal view the body of cingulum.

**Figure 4:**
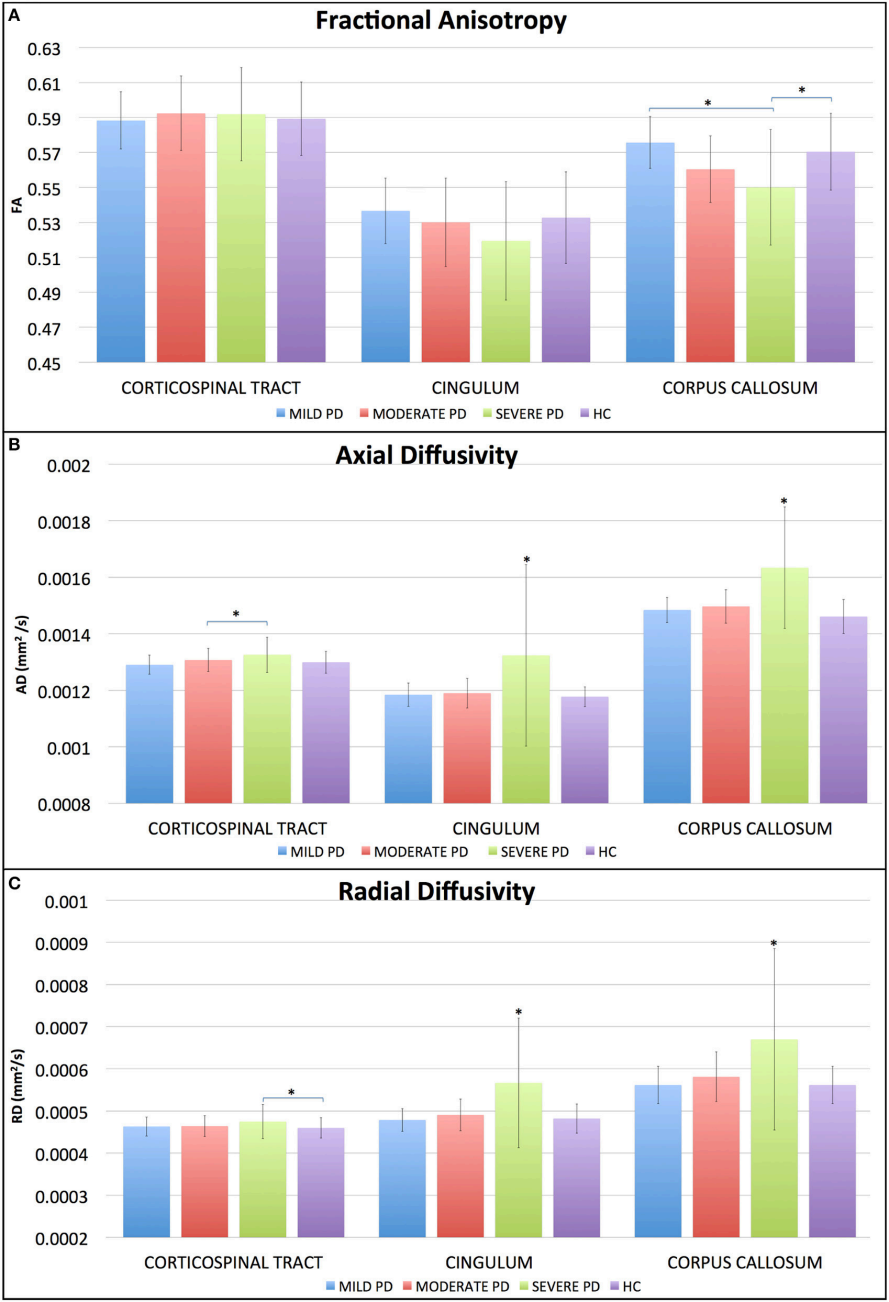
Average diffusion values separated by tracts and groups. SPD patients had lower FA **(A)** in the corpus callosum when compared to HC and MPD. Regarding AD **(B)**, SPD had higher values than MoPD at the CST, and higher values than MPD and HC at cingulum and corpus callosum. RD **(C)** was significant difference (GLM, Sidak's post hoc test, *p* < 0.05). Correlation matrices showing *p* and r values for the corticospinal tract.

There was a positive association between UPDRS-III scores and RD and AD values (*p* < 0.01; Figures 3, 4; see more details in the (Supplemental Material).

## Discussion

We performed two DTI analyzes, TBSS and CST, cingulum and CC tractography. We found diffuse WM abnormalities, indicating significant WM alteration in PD. We also observed differences among patients, and we found an association between clinical scores and diffusion values, showing that some motor and non-motor symptoms relate to specific WM areas.

The results from previous DTI studies in PD are controversial. The main consensus is SN alterations in PD. Regarding other areas, some authors found no differences between PD and HC, while others reported reduced FA, mostly in subcortical regions like the SN pars compacta (SNc) (17–19). Lower FA in SNc and nigrostriatal projection are already present in early disease stages (17, 18); however, FA alterations in the motor, premotor and supplementary motor cortices, cingulum, CC, and CST were also reported (20, 21).

Our TBSS analysis demonstrated WM microstructural differences between PD and HC, mainly in cortico-subcortical pathways. Subcortical abnormalities in PD have been widely discussed, and it was previously believed that the symptoms begin as a result of dopaminergic neurons death in the SN, and that as the disease progresses, the brain involvement ascends to cortical regions (2, 22). More recent studies, however, have demonstrated cortical abnormalities already in early disease stages (14).

We assessed three tracts relevant to PD and focused on all eigenvalues, since DTI analysis is complex the use of only one diffusion variable may not be sufficient to elucidate the mechanisms behind brain alteration. FA is an adimensional value that might not change, even if there are significant abnormalities in AD and RD, as long as these changes are proportional. It is also related with aspects of WM arrangement and density such as myelin integrity, fiber density, and fiber parallelism. Lower FA usually means decreased WM connectivity and has been considered to be an indication of WM microstructural abnormalities. FA is reduced for example when myelin integrity and fiber density have some alteration. Our results showed that it was a sensitive value just for the CC, and just for the SPD group, being lower in this group of patients when compared to HC and MPD. The diffusion analysis of the CC allows the assessment of the degenerative process in inter-hemispheric connectivity, since it transmits sensory, cognitive and motor information through the hemispheres, and alteration in this area, mainly in the body of CC affects complex motor tasks, such as gait (23).

It has been suggested that there is a reduction in WM quantity and quality with age, and as a compensatory strategy older adults need bilateral cortical activation during motor performance. This compensation has been attributed to reduced callosal integrity, diminishing communication between the left and right hemispheres demonstrating the role of cognition in complex motor tasks (24, 25).

The CC is involved in cognitive aspects (26), and we believe this is why only SPD was different from the other groups. In our sample, SPD patients had lower cognitive scores when compared to MPD and MoPD, indicating greater cognitive impairment, and although there was no association between CC diffusion data and SCOPA-COG scores, we found an association between UPDRS-III scores and FA and RD values in CC, corroborating the importance of intact cognition in motricity (25). A recent previous study assessed the relationship between cognitive deficits and motor dysfunction in PD patients, showing that balance skills correlate with executive function, cognitive impairment and the ability to switch attention between two tasks (27).

The CST is the most important efferent tract from M1, and defines the final motor output, that is altered in PD due to altered metabolism and interneuron activity caused by dopamine depletion (20). In our TBSS analysis, only AD was higher in the CST; however, we observed decreased FA and increased AD and RD in the corona radiata, which is a dense WM structure that carries almost all of the neural connections from and to the cerebral cortex and is associated with motor tracts, including the CST.

The ROI analysis of the CST showed that SPD had increased AD and RD in relation to MPD and HC respectively. These findings suggest that CST may not be a pathological target for PD, and corroborates the hypothesis that PD is more complex than just a motor disease.

At the cingulum, TBSS showed increased AD and RD, and ROI analysis confirmed these findings, showing that the SPD had higher AD and RD values comparing to HC. The cingulum is a complex white matter tract, interconnecting cingulate, prefrontal, and temporal cortical areas.

Around 40% of PD patients present some cognitive deficit by the time of diagnosis, and dementia is four times more prevalent in PD than in healthy population (28). The cognitive deficits in PD affect executive function, memory, visuospatial ability, attention, and language (29), and some of these domains are directly involved with the cingulum, such as episodic memory and visuospatial processing and visceral and axial motor control (30). We believe that its degeneration might be responsible for the presence of such nonmotor symptoms in PD (31) and that clinical scales can indirectly measure these abnormalities since we found an association between SCOPA-COG scores and FA and RD values in this region.

In summary, we found diffuse WM abnormalities, including commissural and projection fibers, using the ROI approach; greater alteration was seen in the SPD, even though it was our smaller sample.

Our results support that the WM abnormalities goes beyond the SN and dopaminergic areas, and that areas not directly related to motor skills are also involved, explaining in part some of the non-motor symptoms present in the majority of PD patients. In a previous study, we showed MPD already have cortical involvement (14). This supports the hypothesis that the cortex is already involved in early disease stages. In association with abnormalities found in projection fibers, such as corona radiata, internal and external capsules and corpus callosum, we believe that PD brain alterations are caused by an interruption in integration processes between several brain areas, cortical and subcortical, including gray and white matter abnormalities, as opposed to the previous concept that damage begins in a single cerebral area and would follow a predetermined sequence as disease progresses.

Although PD is a movement disorder, our results, in accordance with previous studies, suggest that the CST seems to not be a pathological target for the disease (32).

We believe this is due to a compensatory mechanism of the brain, where brain alterations are already present, but there are still no clinical symptoms. Thus, we believe that DTI might not be the best tool to assess early abnormalities in PD and that functional alterations may predict structural ones, at least in WM tracts. However, DTI analysis can be used to differentiate disease stages, especially axial and radial tensors, and we believe it could be used as a possible surrogate marker to follow disease progression quantitatively.

We need to address some limitations: this was a cross-sectional study, and we believe a longitudinal approach would be better to investigate disease progression. Also, our findings need to be validated in a larger population, especially regarding disease severity.

## Ethics statement

This study was carried out in accordance with the recommendations of University of Campinas, ethics committee. The protocol was approved by the University of Campinas ethics committee (647/2010). All subjects gave written informed consent in accordance with the Declaration of Helsinki.

## Author contributions

RG, LP, AA-F, and PA contributed to data acquisition and data analysis. RG, BC, and TdR performed statistical analysis. AD and FC were involved in conception and analysis, helped with the interpretation and presentation of the data as well as writing of the article. Research project: RG, AD, and FC conception. RG, AD, and FC organization. RG, LP, PA, BC, TdR, and AA-F execution. Statistical analysis: RG, AD, BC, TdR design. RG, BC, TdR, and AD execution. AD and FC review and critique. RG manuscript preparation. RG writing of the first draft. AD and FC review and critique.

### Conflict of interest statement

The authors declare that the research was conducted in the absence of any commercial or financial relationships that could be construed as a potential conflict of interest.

## References

[1] Daniel SE Lees AJ. Parkinson's Disease Society Brain Bank, London: overview and research. J Neural Transm Suppl. (1993) 39:165–72. 8360656

[2] Braak H Del Tredici K Rub U de Vos RA Jansen Steur EN Braak E. Staging of brain pathology related to sporadic Parkinson's disease. Neurobiol Aging (2003) 24:197–211. 10.1016/S0197-4580(02)00065-9 12498954

[3] Beach TG Adler CH Lue L Sue LI Bachalakuri J Henry-Watson J. Unified staging sys-tem for Lewy body disorders: correlation with nigrostriatal degeneration, cognitive impairment and mo-tor dysfunction. Acta Neuropathol. (2009) 117:613–34. 10.1007/s00401-009-0538-8 19399512 PMC2757320

[4] Sauerbier A Jenner P Todorova A Chaudhuri KR Non motor subtypes and Parkinson's dis-ease. Parkinsonism Relat Disord. (2016) 22(Suppl. 1):S41–6. 10.1016/j.parkreldis.2015.09.027 26459660

[5] Rocca WA Bower JH Ahlskog JE Elbaz A Grossardt BR McDonnell SK. Risk of cogni-tive impairment or dementia in relatives of patients with Parkinson disease. Arch Neurol. (2007) 64:1458–64. 10.1001/archneur.64.10.1458 17923629

[6] Leverenz JB Quinn JF Zabetian C Zhang J Montine KS Montine TJ. Cognitive impairment and dementia in patients with Parkinson disease. Curr Top Med Chem. (2009) 9:903–12. 19754405 PMC2804995

[7] Gattellaro G Minati L Grisoli M Mariani C Carella F Osio M. White matter involvement in idiopathic Parkinson disease: a diffusion tensor imaging study. AJNR Am J Neuroradiol. (2009) 30:1222–6. 10.3174/ajnr.A1556 19342541 PMC7051338

[8] Skidmore FM Yang M Baxter L von Deneen KM Collingwood J He G. Reliability anal-ysis of the resting state can sensitively and specifically identify the presence of Parkinson disease. Neuroimage (2013) 75:249–61. 10.1016/j.neuroimage.2011.06.056 21924367

[9] Mori S Zhang JY Principles of diffusion tensor imaging and its applications to basic neurosci-ence research. Neuron (2006) 51:527–39. 10.1016/j.neuron.2006.08.012 16950152

[10] Alexander AL Lee JE Lazar M Field AS. Diffusion tensor imaging of the brain. Neurothera-peutics (2007) 4:316–29. 10.1016/j.nurt.2007.05.011 17599699 PMC2041910

[11] Atkinson-Clement C Pinto S Eusebio A Coulon O. Diffusion tensor imaging in Parkinson's disease: review and meta-analysis. Neuroimage Clin. (2017) 16:98–110. 10.1016/j.nicl.2017.07.011 28765809 PMC5527156

[12] Saeed U Compagnone J Aviv RI Strafella AP Black SE Lang AE. Imaging biomarkers in Parkinson's disease and Parkinsonian syndromes: current and emerging concepts. Transl Neuro-degener (2017) 6:8. 10.1186/s40035-017-0076-6 28360997 PMC5370489

[13] Malek N Lawton MA Swallow DM Grosset KA Marrinan SL Bajaj N. Vascular disease and vascular risk factors in relation to motor features and cognition in early Parkinson's disease. Mov Disord. (2016) 31:1518–26. 10.1002/mds.26698 27324570 PMC5082556

[14] Guimaraes RP Arci Santos MC Dagher A Campos LS Azevedo P Piovesana LG Pat-tern of reduced functional connectivity and structural abnormalities in Parkinson's disease: an Ex-ploratory study. Front Neurol. (2016) 7:243 10.3389/fneur.2016.00243 28133455 PMC5233672

[15] Smith SM Jenkinson M Johansen-Berg H Rueckert D Nichols TE Mackay CE. Tract-based spatial statistics: voxelwise analysis of multi-subject diffusion data. Neuroimage (2006) 31:1487–505. 10.1016/j.neuroimage.2006.02.024 16624579

[16] Campos BM Coan AC Beltramini GC Liu M Yassuda CL Ghizoni E White matter ab-normalities associate with type and localization of focal epileptogenic lesions. Epilepsia (2015) 56:125–32. 10.1111/epi.12871 25545559

[17] Yoshikawa K Nakata Y Yamada K Nakagawa M. Early pathological changes in the parkin-sonian brain demonstrated by diffusion tensor MRI. J Neurol Neurosurg Psychiatry (2004) 75:481–4. 10.1136/jnnp.2003.021873 14966170 PMC1738942

[18] Chan LL Rumpel H Yap K Lee E Loo HV Ho GL. Case control study of diffusion tensor imaging in Parkinson's disease. J Neurol Neurosurg Psychiatry (2007) 78:1383–6. 10.1136/jnnp.2007.121525 17615165 PMC2095589

[19] Vaillancourt DE Spraker MB Prodoehl J Abraham I Corcos DM Zhou XJ. High-resolution diffusion tensor imaging in the substantia nigra of de novo Parkinson disease. Neurology (2009) 72:1378–84. 10.1212/01.wnl.0000340982.01727.6e 19129507 PMC2677508

[20] Zhan W Kang GA Glass GA Zhang Y Shirley C Millin R. Regional alterations of brain microstructure in Parkinson's disease using diffusion tensor imaging. Mov Disord. (2012) 27:90–7. 10.1002/mds.23917 21850668 PMC4472452

[21] Lenfeldt N Hansson W Larsson A Nyberg L Birgander R Forsgren L Diffusion tensor imag-ing and correlations to Parkinson rating scales. J Neurol. (2013) 260:2823–30. 10.1007/s00415-013-7080-2 23974647

[22] Braak H Bohl JR Muller CM Rub U de Vos RA Del Tredici K. Stanley Fahn Lecture 2005: the staging procedure for the inclusion body pathology associated with sporadic Parkinson's disease reconsidered. Mov Disord. (2006) 21:2042–51. 10.1002/mds.21065 17078043

[23] Fling BW Dale ML Curtze C Smulders K Nutt JG Horak FB. Associations between mobility, cognition and callosal integrity in people with parkinsonism. Neuroimage Clin. (2016) 11:415–22. 10.1016/j.nicl.2016.03.006 27104136 PMC4827724

[24] Fling BW Peltier SJ Bo J Welsh RC Seidler RD Age differences in interhemispheric interac-tions: callosal structure, physiological function, and behavior. Front Neurosci. (2011) 5:38 10.3389/fnins.2011.00038 21519384 PMC3077973

[25] Wang M Jiang S Yuan Y Zhang L Ding J Wang J. Alterations of functional and struc-tural connectivity of freezing of gait in Parkinson's disease. J Neurol. (2016) 263:1583–92. 10.1007/s00415-016-8174-4 27230857

[26] Goldman JG Bledsoe IO Merkitch D Dinh V Bernard B Stebbins GT. Corpus callosal atro-phy and associations with cognitive impairment in Parkinson disease. Neurology (2017) 88:1265–72. 10.1212/WNL.0000000000003764 28235816 PMC5373777

[27] Varalta V Picelli A Fonte C Amato S Melotti C Zatezalo V. Relationship between Cog-nitive performance and motor dysfunction in patients with Parkinson's disease: a pilot cross-sectional study. Biomed Res Int. (2015) 2015:365959. 10.1155/2015/365959 25918713 PMC4396143

[28] Williams-Gray CH Evans JR Goris A Foltynie T Ban M Robbins TW. The distinct cog-nitive syndromes of Parkinson's disease: 5 year follow-up of the CamPaIGN cohort. Brain (2009) 132(Pt 11):2958–69. 10.1093/brain/awp245 19812213

[29] McKinlay A Grace RC Dalrymple-Alford JC Roger D. Cognitive characteristics associated with mild cognitive impairment in Parkinson's disease. Dement Geriatr Cogn Disord. (2009) 28:121–9. 10.1159/000235247 19690414

[30] Vogt BA Nimchinsky EA Vogt LJ Hof PR. Human cingulate cortex: surface features, flat maps, and cytoarchitecture. J Comp Neurol. (1995) 359:490–506. 10.1002/cne.903590310 7499543

[31] Kamagata K Motoi Y Abe O Shimoji K Hori M Nakanishi A. White matter alteration of the cingulum in Parkinson disease with and without dementia: evaluation by diffusion tensor tract-specific analysis. AJNR Am J Neuroradiol. (2012) 33:890–5. 10.3174/ajnr.A2860 22241380 PMC7968830

[32] Lu MK Chen CM Duann JR Ziemann U Chen JC Chiou SM Investigation of motor cortical plasticity and corticospinal tract diffusion tensor imaging in patients with Parkinsons Dis-ease and essential tremor. PLoS ONE (2016) 11:e0162265 10.1371/journal.pone.0162265 27603204 PMC5014415

