# Supplementary figures and images for: Is Diffusion Tensor Imaging a Good Biomarker for Early Parkinson's Disease?
Source: Front Neurol. 2018 Aug 21;9:626. doi: 10.3389/fneur.2018.00626 (PMC6111994; doi:10.3389/fneur.2018.00626)

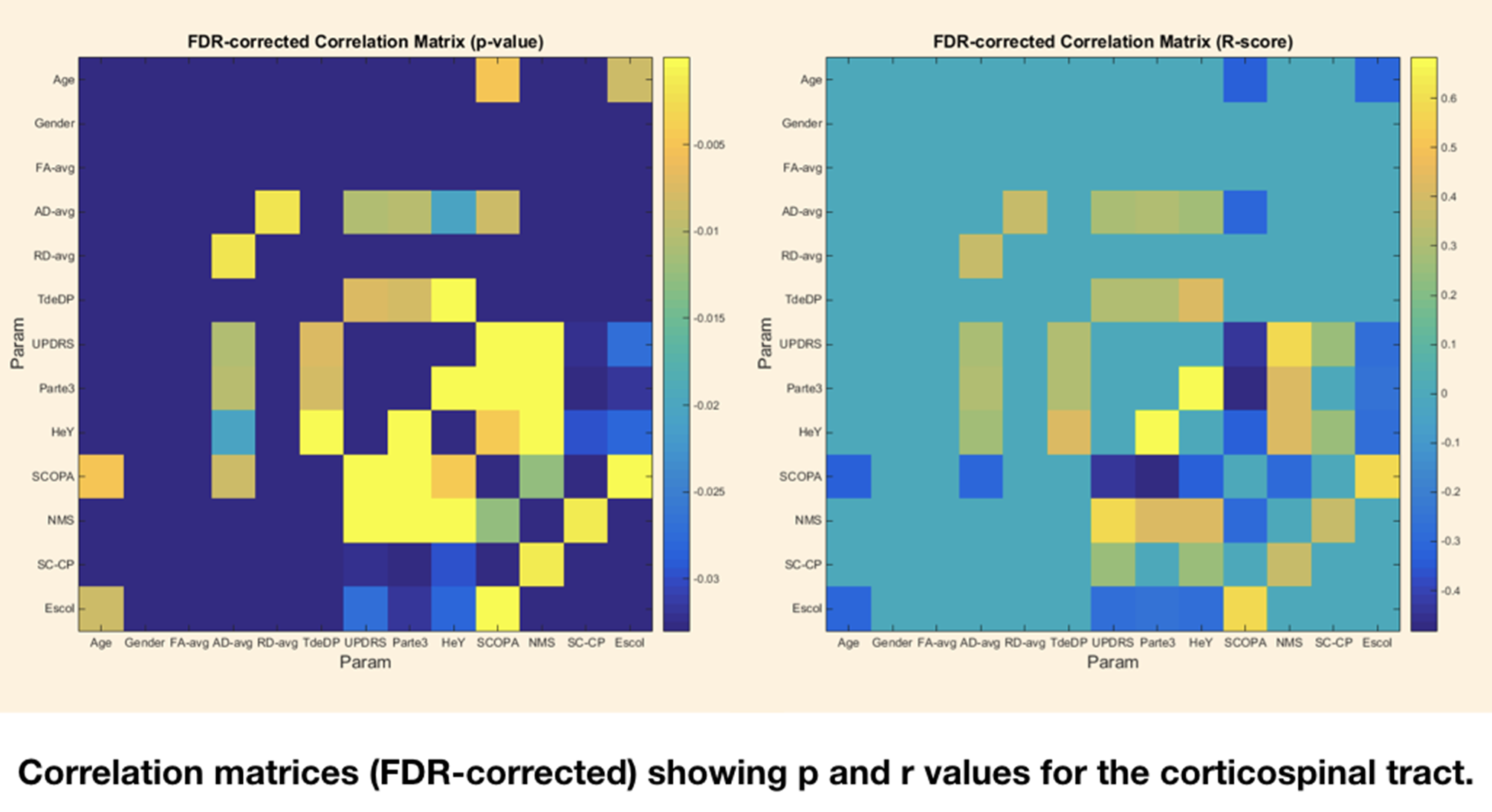

Supplement: Supplementary file 1 [file Image_1.tiff]

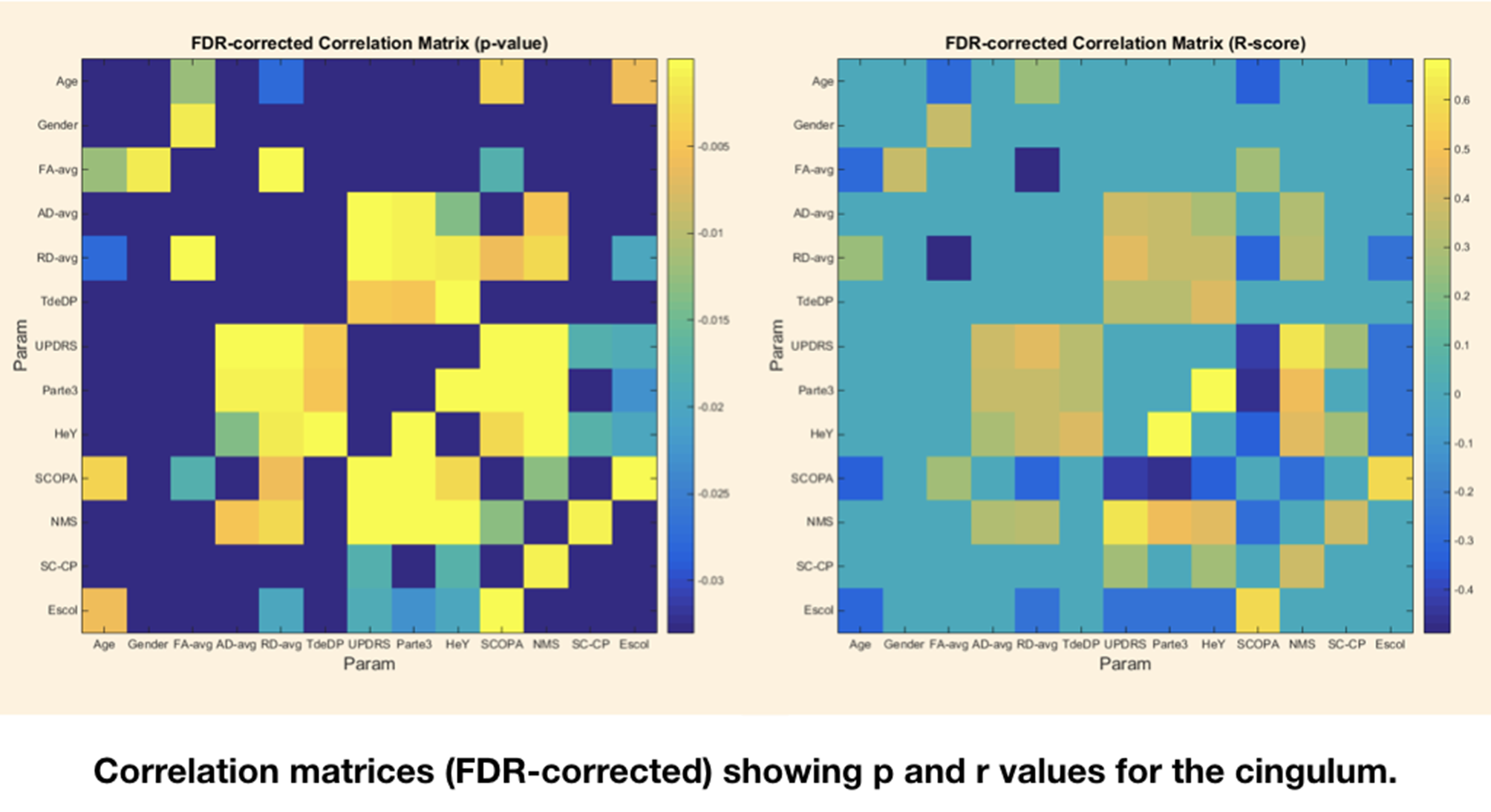

Supplement: Supplementary file 2 [file Image_2.tiff]

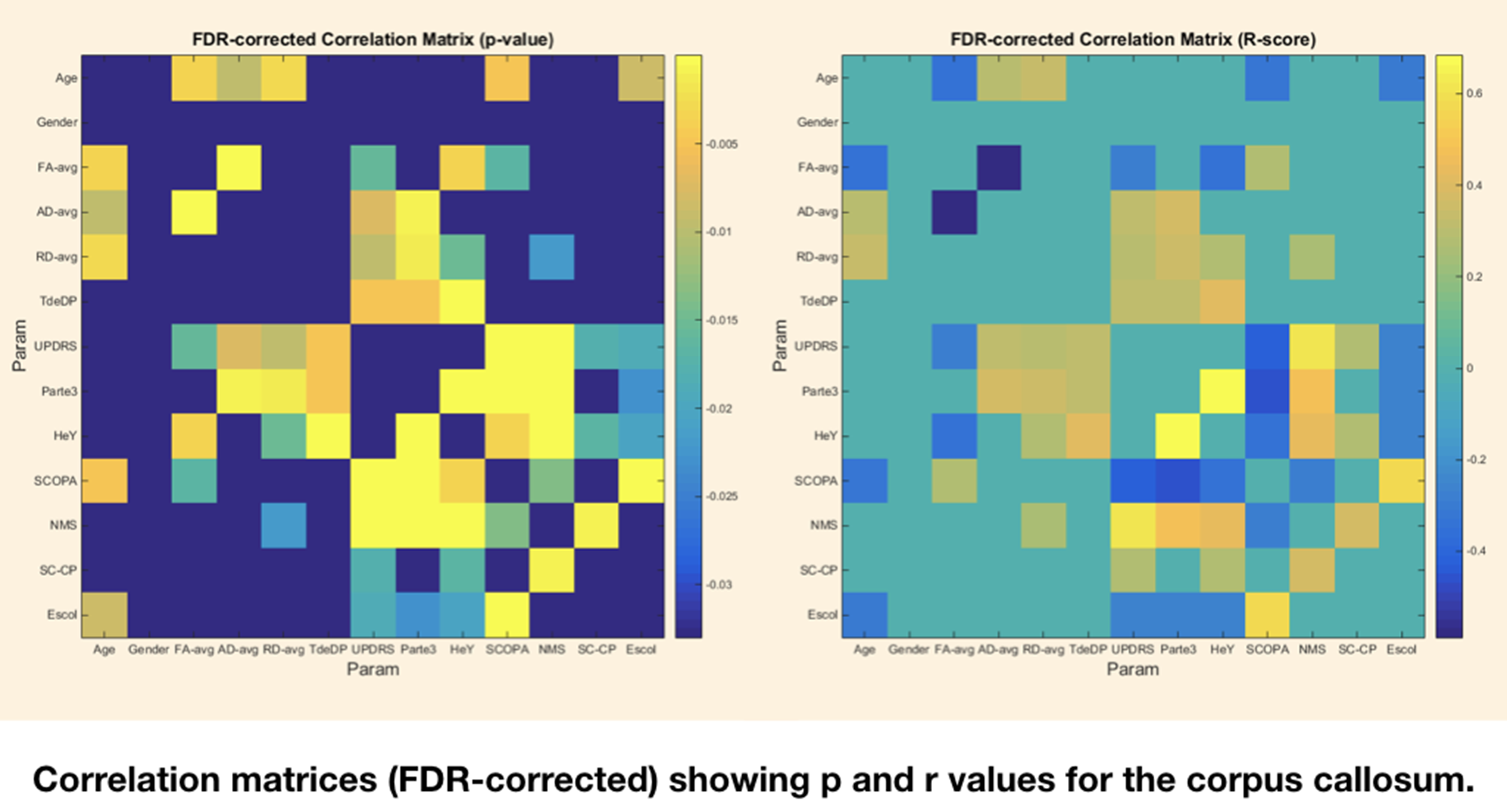

Supplement: Supplementary file 3 [file Image_3.tiff]
